# Real-world outcomes of chemotherapy plus immune checkpoint inhibitors versus chemotherapy alone in advanced, unresectable, and recurrent intrahepatic cholangiocarcinoma

**DOI:** 10.3389/fimmu.2024.1390887

**Published:** 2024-05-23

**Authors:** Tinotenda Blessing Madzikatire, Shan Heng, HongYi Gu, YunFeng Shan, EnHua Lin, Joshua Banda, Asta Debora, Brandon Anotida Madziva, Mutale Jaeste Bowa, Munyaradzi Godfrey Mudhuri, Canol Bwalya

**Affiliations:** ^1^ Department of Hepatobiliary Surgery, The First Affiliated Hospital of Wenzhou Medical University, Wenzhou, Zhejiang, China; ^2^ Wenzhou Medical University, Wenzhou, Zhejiang, China

**Keywords:** advanced unresectable, recurrent, intrahepatic cholangiocarcinoma (iCCA), immunotherapy, chemotherapy, combination (combined) therapy

## Abstract

**Background:**

There are limited treatment options available to improve the prognosis of patients with advanced or metastatic cholangiocarcinoma particularly intrahepatic cholangiocarcinoma (iCCA). This study aimed to evaluate the efficacy and safety of combining chemotherapy plus anti-PD-1/L1 drugs compared to chemotherapy alone in advanced, unresectable, and recurrent intrahepatic cholangiocarcinoma patients.

**Methods:**

Patients with advanced, unresectable, or recurrent iCCA who received chemotherapy combined with PD-1/PD-L1 inhibitors or chemotherapy alone were retrospectively screened and analyzed. The primary outcomes were overall survival (OS) and progression-free survival (PFS). The secondary outcomes were overall response rate (ORR), disease control rate (DCR), and safety.

**Results:**

81 eligible patients were included in the study (chemotherapy plus anti-PD-1/L1 group n=51, and chemotherapy-alone group n=30). The median OS was 11 months for the chemotherapy plus anti-PD-1/L1 group, significantly longer than the 8 months in the chemotherapy-alone group, with a hazard ratio (HR) of 0.53 (95% CI 0.30–0.94, *P* = 0.008). The median PFS of 7 months in the chemotherapy plus anti-PD-1/L1 group was significantly longer than the 4 months in the chemotherapy-alone group, with HR of 0.48 (95% CI 0.27–0.87); *P* = 0.002). Similarly, the combined therapy group showed a higher ORR (29.4%) and DCR (78.4%) compared to 13.3% and 73.3% in the chemotherapy-alone group, respectively. More grade 3–4 treatment-related adverse effects were recorded in the chemotherapy plus anti-PD-1/L1 group (66.7%) compared to the chemotherapy-alone group (23.3%), however, they were manageable and tolerable.

**Conclusion:**

Chemotherapy plus anti-PD-1/L1 represents a more effective and tolerable treatment option for advanced, unresectable, and recurrent iCCA patients compared to chemotherapy alone.

## Introduction

Cholangiocarcinoma (CCA) is a type of biliary tract cancer (BTC) that constitutes a cluster of malignancies originating from the epithelium of the biliary tree (1). CCAs are divided into two major groups, intrahepatic CCA (iCCA) and extrahepatic CCA (eCCA). The eCCA group is further divided into the perihilar (pCCA) and distal (dCCA) (1–4). Globally, CCA is the second most common primary liver cancer after hepatocellular carcinoma (HCC). Approximately, 3% of all global gastrointestinal malignancies are CCAs, of which eCCAs account for at least 80% while the remaining cases are iCCA (5). Common risk factors for CCAs include Primary sclerosing cholangitis (PSC), Opisthorchis viverrini and Clonorchis sinensis parasite infection, choledochal cysts (CCs), hepatitis B virus (HBV), and hepatitis C virus (HCV) (6–8). The global rise in risk factors, such as alcohol and tobacco use, metabolic conditions, as well as viral infection, may account for the recently observed rise in CCA cases and the increased mortality rate (9). As accurate diagnosis is difficult, most CCA cases, especially iCCA cases, are identified in an advanced stage, which limits treatment options.

Surgery is the primary and most effective treatment option for CCAs, particularly in patients with early-stage iCCA (2). However, the survival rate post-surgery is low as complete resection is often unattainable. For instance, in iCCA, the 5-year survival rate post-resection is 24%, and nearly 60–70% of patients who undergo resection experience recurrence (10). As such, in patients with incomplete resection or reoccurring, palliative chemotherapy is administered to manage downstaging and control metastasis. Chemotherapy is the primary treatment option for patients with advanced, unresectable, or metastasized CCA (11). Chemotherapy regimens used in CCA include gemcitabine and cisplatin (GemCis), gemcitabine plus S-1 (GS), gemcitabine and oxaliplatin (GEMOX), and a combination of oxaliplatin, irinotecan, and infusional fluorouracil (mFOLFIRINOX) (12, 13). Alternative treatment options include the use of immune checkpoint inhibitors (ICI). Specifically, programmed cell death protein 1 (PD-1) inhibitors have been tested in advanced and unresectable CCA (2). ICI monotherapy has yielded subpar results with low progression-free survival (PFS) and overall survival (OS) (14). Although not fully elucidated, data from studies utilizing the combination of ICI and chemotherapy suggest that combination therapy yields better outcomes (15, 16). However, extensive investigations are required to assess the efficacy of chemotherapy plus ICI, especially in clinical settings.

To date, few publications have demonstrated the efficacy of integrating ICI and chemotherapy in iCCA. Existing data evaluates advanced BTC patients often comprising of a small iCCA sample size. Compared to other BTCs, iCCA has a unique molecular characteristic, presentation, prognosis, and different management and treatment, thus, data derived from these studies may not be a true representation of the efficacy of combination therapy, within the context of clinical intervention.

In light of this, we conducted a retrospective investigation evaluating the clinical efficacy and safety of combining chemotherapy with PD-1/L1 in iCCA patients diagnosed with unresectable, metastatic, and recurrent iCCA. We believe that the combination of ICI and chemotherapy yields beneficial survival outcomes for advanced iCCA patients.

## Methods

### Study design and patients

We conducted an institutional review board-approved retrospective study on advanced unresectable and recurrent iCCA patients treated at the First Affiliated Hospital of Wenzhou University between December 1, 2019, and April 30, 2023. This study followed Good Clinical Practice guidelines and the Declaration of Helsinki ethical principles.

The inclusion criteria were as follows (1): patients older than 18 years with histopathologically confirmed unresectable or metastatic iCCA, (2) patients who had at least one measurable tumor lesion at baseline per the Response Evaluation Criteria 1.1 in Solid Tumors (RECIST criteria 1.1), (3) Patients who were treated with either systemic chemotherapy or chemotherapy plus PD-1/L1, (4) patients who had Child-Pugh class A or B liver function status (score ≤7), (5) Patients who presented with an Eastern Cooperative Oncology Group performance status (ECOG PS) value of 0–1(Using a scale from 0 to 5, where lower scores indicate higher levels of functioning).

Exclusion criteria were set for individuals with severely impaired liver function, specifically those of Child-Pugh class C. Patients who did not receive more than one treatment regimen, either a combination therapy or chemotherapy-alone. The patients who had additional coexisting malignant tumors, patients who did not have any measurable tumors on both computerized tomography (CT) and magnetic resonance imaging (MRI), and patients with incomplete data.

### Data collection

All pathological diagnosis data were obtained from the electronic system of the hospital’s pathology department, whilst all clinical data and treatment histories were independently extracted from both the hospitals’ inpatient and outpatient electronic systems. Current picture archiving computer software (PACS) system was used to obtain electronic linear, as well as circumferential tracings of tumors on either CT or MRI scans, images were regularly evaluated every two to three months during treatment. Tumor measurement data were independently reviewed and assessed by two radiologists per the RECIST criteria 1.1. Patient demographic and basic characteristics including age, gender, BMI, HBV, HCV, CA19–9 levels, AFP levels, Child-Pugh, ECOG score, tumor number, tumor differentiation, and recurrence were obtained and recorded in (**Table 1**). Data was last updated on April 30, 2023. Written informed consent was waived since this study was conducted retrospectively, however, the patients’ identities were anonymized to ensure their confidentiality. Patients who did not have any documented deaths were censored from the analysis at the cut-off date, April 30, 2023.

**Table 1:** Baseline characteristics and demographics of the entire and subgroup study population.

Characteristic	Chemo plus anti-PD-1/L1 (n=51)	Chemo alone (n=30)	P value
**Gender**			0.708
Male	25 (49)	16 (53.3)	
Female	26 (51)	14 (46.7)	
**Age**			0.260
<63 years	27 (52.9)	12 (40.0(	
>63 years	24 (47.1)	18 (60)	
**BMI**			0.044
Normal	35 (68.6)	19 (63.3)	
Obese	15 (28.6)	6 (20.0)	
Overweight	1 (2.0)	5 (16.7)	
**HBV**	9 (19.8)	7 (23.3)	0.572
**HCV**	0	0	
**CA19–9 levels**			0.474
<200	23 (45.1)	16 (53.3)	
>200	28 (54.9)	14 (46.7)	
**AFP levels**			0.311
Normal	42 (82.4)	27 (90.0)	
High	7 (17.6)	3 (10.0)	
**Child-Pugh**			0.474
Class A	46 (90.2)	28 (93.3)	
Class B	5 (9.8)	2 (6.7)	
**ECOG score**			0.313
0	17 (34.0))	7 (23.3)	
1	33 (66.0)	23 (76.7)	
**Tumor number**			0.011
<2	22 (45.1)	15 (50)	
>2	28 (54.9)	15 (50)	
**Differentiation**			0.116
poor	19 (33.3)	10 (37.3)	
moderate	12 (23.5)	10 (33.3)	
High	5 (9.8)	2 (2.4)	
N/A	13(25.5)	6 (20.0)	
Site of Metastasis			
Liver	49 (96.1)	27 (90.0)	0.005
Lymph node	34 (66.7)	25 (83.3)	0.003
Lung	11 (21.6)	3 (10.0)	0.007
Bone	14 (27.5)	4 (13.3)	0.003
**Recurrent**	12 (23.5)	18 (60.0)	

Data in () are %. BMI, body mass index; HBV, hepatitis B virus; HCV, hepatitis C virus; CA 19–9, carbohydrate antigen 19.9; AFP, alpha-fetoprotein; ECOG, Eastern Cooperative Oncology Group.

### Study outcomes and objectives

The primary outcome evaluated was overall survival (OS), which is defined as the duration from the commencement of treatment to the occurrence of death due to any cause compared between the chemotherapy combined with PD-1/L1 therapy group versus the chemotherapy-alone group. Progression-free survival (PFS), was defined as the duration from the initiation of treatment to the occurrence of disease progression or death from any cause between the combined therapy group and the chemotherapy group. Additionally, secondary endpoints included the objective response rate (ORR), measuring the percentage of patients who had a confirmed complete or partial response according to RECIST version 1.1, and the disease control rate (DCR), measuring the proportion of patients who achieved a complete or partial response or stable disease according to RECIST version 1.1.

Safety was additionally assessed as a secondary outcome, with all adverse events reported by patients or clinicians recorded by the National Cancer Institute Common Terminology Criteria for Adverse Events, version 5.0. The study was conducted per the Transparent Reporting of Evaluations with Nonrandomized Designs (TREND) statement checklist.

### Statistical analysis

The baseline characteristics and efficacy data of the two treatment groups were compared using either the Chi-square test or Fisher’s exact test. Survival analyses to generate PFS and OS curves were conducted via the Kaplan-Meier method. The Log-rank test was used to determine the P value and compare curves from the two treatment groups. Hazards ratios were estimated using the Cox proportional hazards model. The statistical significance was set at P<0.05. The statistical analyses were conducted using SPSS 20.0 software (IBM, SPSS, Chicago, IL, USA) and GraphPad Prism, Version 8.2.0 (GraphPad, Inc.).

## Results

### Patient characteristics

**Figure 1:**
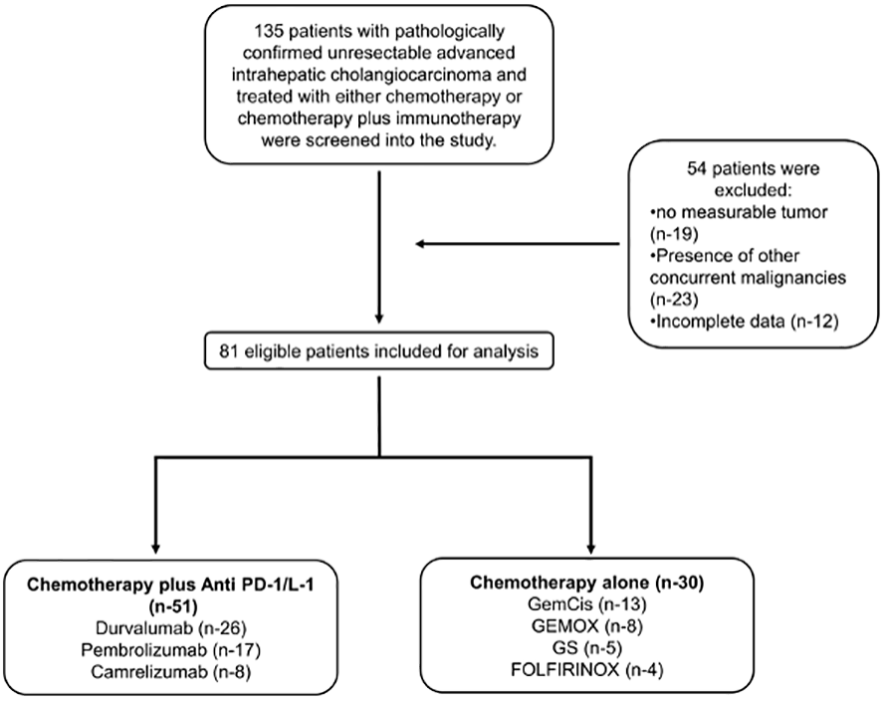
Flow chart of study design.

Between December 2019 and April 2023, 135 patients had been diagnosed with advanced or recurrent iCCA and had received either chemotherapy alone or combined with PD-1/L1. Of the 135 patients, 54 patients were excluded based on the eligibility criteria leaving 81 patients for analysis. Two cohort groups were formed with the following sample size: 51 in the combined therapy (chemotherapy plus PD-1/L1) group and 30 in the chemotherapy-alone group (**Figure 1**). The combined therapy group consisted of patients treated with chemotherapy plus; durvalumab (26 patients); pembrolizumab (17 patients); and camrelizumab (8 patients) (**Supplementary Table S1**). Patients in the chemotherapy group were treated with; GemCis (13 patients); gemcitabine and oxaliplatin (GEMOX) (8 patients); gemcitabine and S-1 (GS) (5 patients); FOLFIRINOX (4 patients) (**Supplementary Table S2**).

Patient demographics and baseline characteristics were recorded (**Table 1**). Patients consisted of 50.6% (n = 41) males and 49.4% (n = 40) females with a median age of 63 years (range 29–89 years). Most patients had a normal BMI and an ECOG score of 1. 16 patients had HBV while none had HCV. Furthermore, 29 patients had poorly differentiated tumors while 22 had moderately differentiated tumors. Liver and lymph nodes were the most frequent metastatic sites across all patients. There was an insignificant difference between the number of patients that had recurrent iCCA post-resection in the combined therapy group (12 patients) and the chemotherapy-alone group (18 patients).

### Efficacy

**Figure 2:**
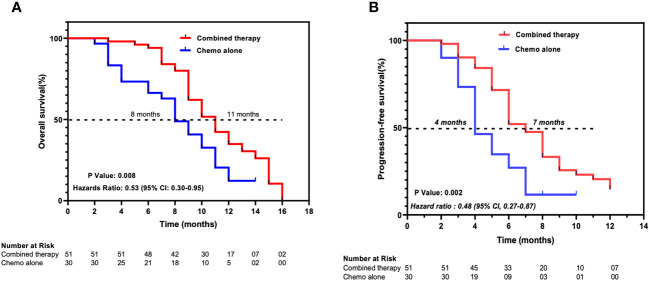
Kaplan-Meier estimates of **(A)** overall survival and **(B)** progression-free survival. Combined therapy, chemotherapy plus ant-PD-1/L1 group; Chemo alone, Chemotherapy-alone group. Ticks represent censored patients.

As of April 30, 2023, 62 (76.5%) deaths had occurred, 38 (74.5%) patients in the combined therapy group and 24 (80%) patients in the chemotherapy-alone group. The median OS was 11 months (95% CI 9.57–12.43) for the patients in the combined therapy group and 8 months (95% CI 6.34–9.64) for those in the chemotherapy-alone group. The hazard ratio (HR) for OS was 0.53 (95% CI 0.30–0.94, *P* = 0.008) for the combined therapy group versus the chemotherapy-alone group. The median PFS was significantly longer in the combined therapy group at 7 months (95% CI 5.88–8.12) compared to the chemotherapy-alone group at 4 months, (95% CI 3.027–4.97). The HR for PFS of the combined therapy cohort versus the chemotherapy-alone cohort was 0.48 (95% CI 0.27–0.87, *P* = 0.002) (**Figure 2**).

**Figure 3:**
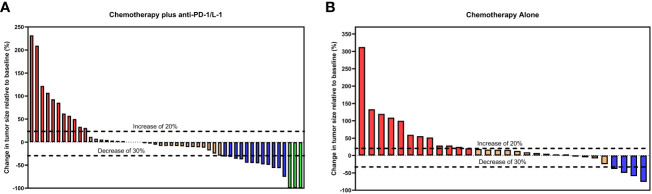
Waterfall plots for tumor size change relative to baseline size in the **(A)** chemotherapy plus ant-PD-1/L1 group and **(B)** chemotherapy-alone group. Red bars represent progressive disease (PD), orange bars represent stable disease (SD), blue bars represent partial response (PR) and green bars represent complete response (CR) per RECIST Criteria 1.1.

More patients attained radiologically confirmed CR and PR in the combined therapy group as compared to the chemotherapy-alone group, 5.9% (n = 3/51) versus 0% and 23.5% (n = 12/51) versus 13.3% (n = 4/30) respectively. Patients in the combined therapy group exhibited a significantly higher ORR (CR + PR), 29.4% (n = 15/51), compared to those in the chemotherapy-alone group, 13.3% (n = 4/30). Similarly, the combined therapy group had a higher DCR (CR+PR+SD) when compared to the chemotherapy-alone group, 78.4% (n = 40/51) and 73.3% (n = 22/30) respectively. 3 of the 12 patients who achieved PR in the combined therapy group underwent curative tumor resection following a preoperative evaluation (**Table 2**). Overall, patients in the combined therapy group showed a better tumor percentage change relative to baseline size compared to the patients in the chemotherapy-alone group (**Figure 3**).

**Table 2:** Therapeutic efficacy of response outcomes in both groups.

	Chemotherapy plus PD-1/L1 (n-51)	Chemotherapy-alone (n-30)
Best Overall Response		
Complete Response (CR)	3 (5.9%)	0 (0%)
Partial Response (PR)	12 (23.5%)	4 (13.3%)
Progressive Disease (PD)	11 (21.6%)	8 (26.7%)
Stable Response (SD)	25 (49.0%)	18 (60%)
**Objective Response Rate (ORR)**	29.4%	13.3%
**Disease Control Rate (DCR)**	78.4%	73.3%

### Safety and tolerability

Treatment-related adverse effects (TRAEs) occurred in 98.0% (50 of 51) of the patients from the combined therapy group while 90.0% (27 of 30) of the patients in the chemotherapy-alone group experienced TRAEs. Nausea and leukopenia were the predominant grade 1–2 TRAEs in the combined therapy group, 37.3% and 33.3% respectively, while anemia (20%) and leukopenia (16.7%) were predominant in the chemotherapy-alone group. Malaise (7.8%), vomiting (3.9%), and diarrhea (2%) were only recorded in the combined therapy group whereas elevated AST (10%) was only recorded in the chemotherapy-alone group (**Table 3**). The chemotherapy-alone group had fewer grade 3–4 TRAEs, 7 of 30 (23.3%) patients, compared to the combined therapy group, 34 of 51 (66.7%) patients. The most frequent grade 3–4 TRAEs were leukopenia (17.6% in the combined therapy group and 3.3% in the chemotherapy-alone), thrombocytopenia (13.7% in the combined therapy group and 10.0% in the chemotherapy-alone), and elevated bilirubin levels (9.8% in the combined therapy group and 6.7% in the chemotherapy-alone). The elevated bilirubin levels, AST levels, and ALT levels were likely due to disease progression of lesions occupying the bile ducts. In both groups, no patient discontinued therapy due to any TRAEs and no cases of severe adverse events or treatment-related deaths occurred.

**Table 3:** Treatment-related adverse effects experienced in both groups.

	Chemotherapy plus PD-1/L1 (n-51)		Chemotherapy-alone (n-30)	
Any grade	49 (98.0%)		27(90.0%)	
**Adverse Effect**	**Grade1–2**	**Grade 3–4**	**Grade 1–2**	**Grade 3–4**
Malaise	4 (7.8%)	0	0	0
Pruritus	3 (5.9%)	0	1 (3.3%)	0
Nausea	19 (37.3%)	1 (2%)	2 (6.7%)	0
Vomiting	2 (3.9%)	0	0	0
Diarrhea	1 (2%)	0	0	0
Fatigue	8 (15.6%)	1 (2%)	2 (6.7%)	0
Chest pain	3 (5.9%)	0	1 (3.3%)	0
Anemia	9 (17.6%)	5 (9.8%)	6 (20%)	1 (3.3%)
Fever	5 (9.8%)	0	2 (6.7%)	0
Abdominal pain	3 (5.9%)	0	2 (6.7%)	0
Leukopenia	17 (33.3%)	15 (17.6%)	5 (16.7%)	1 (3.3%)
thrombocytopenia	9 (17.6%)	7 (13.7%)	4 (13.3%)	3 (10%)
Constipation	5 (9.8%)	0	1 (3.3%)	0
Elevated bilirubin levels	4 (7.8%)	5 (9.8%)	4 (13.3%)	2 (6.7%)
Elevated AST	0	0	3 (10%)	0
Elevated ALT	5 (9.8%)	0	3 (10%)	

*AST- aspartate aminotransferase.

*ALT- alanine aminotransferase.

### Subgroup analysis

**Figure 4:**
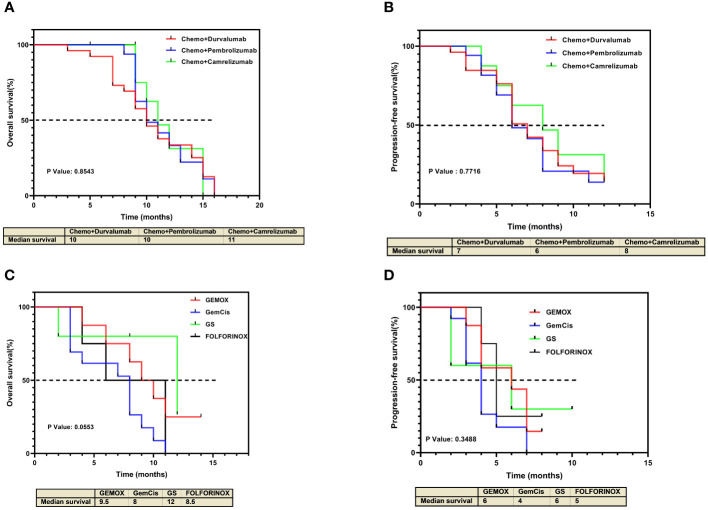
Kaplan-Meier estimates of overall survival and progression-free survival for individual treatment regimens. **(A)** Overall survival and **(B)** progression-free survival for individual treatment regimens in the chemotherapy plus anti-PD-1/L1 group. **(C)** Overall survival and **(D)** progression-free survival for individual treatment regimens in the chemotherapy-alone group.

In the combined therapy group, comparing the various anti-PD-1/L-1 drugs used, revealed no significant differences in either OS or PFS (P values = 0.8543 and = 0.6221, respectively) (**Figures 4A, B**). Similarly, no significant difference was observed in both OS and PFS in the various chemotherapy regimens used in the chemotherapy-alone group (P values = 0.0553 and = 0.3488, respectively) (**Figures 4C, D**). However, in the chemotherapy-alone group, patients treated with the GS regimen exhibited a higher OS (12 months) compared to the other regimens (GEMOX 9.5 months, GemCis 8 months, and FOLFORINOX 8.5 months) (**Figure 4C**).

**Figure 5:**
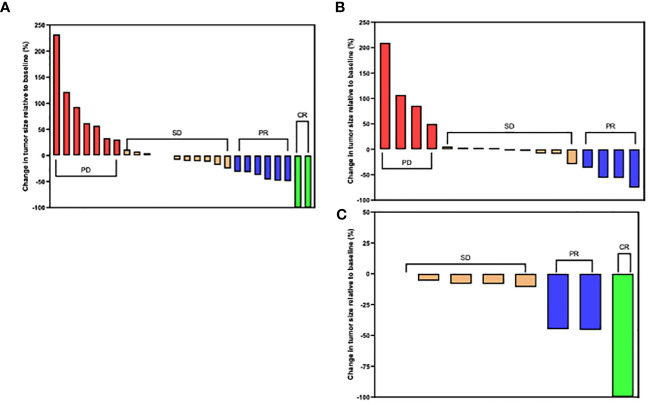
Tumor size changes relative to baseline size in the chemotherapy plus anti-PD-1/L1 group. **(A)** Chemotherapy plus Durvalumab. **(B)** Chemotherapy plus Pembrolizumab. **(C)** Chemotherapy plus Camrelizumab. (CR) complete response; (PR) partial response; (SD) stable disease and (PD) progressive disease per RECIST Criteria 1.1.

In the combined therapy group, 3 patients achieved CR. Of these 3, 2 patients were treated with chemotherapy plus durvalumab while 1 was treated with chemotherapy plus camrelizumab (**Figures 5A, C**). Additionally, of patients treated with chemotherapy plus durvalumab 6 patients achieved PR, 11 had SD, and 7 had PD. In the chemotherapy plus pembrolizumab subgroup, 4 patients had PD, 9 had SD, and 4 achieved PR (**Figure 5B**). Of the patients treated with chemotherapy plus camrelizumab 2 achieved PR and 4 had SD. Interestingly all patients (n = 7) treated with chemotherapy plus camrelizumab had no tumor progression (**Figure 5A**).

## Discussion

BTC are rare and highly aggressive tumors that exhibit a diminished rate of response and dismal prognosis when subjected to treatment (17). From the results from the ABC-02 trial, when unresectable, metastatic, or recurring, chemotherapy has been established as the first-line treatment method for BTC (12). Recently, the use of ICI and chemotherapy has gained ground due to its ability to extend OS and PFS in various types of tumors such as non-small cell lung cancer and renal cell carcinoma (18). The use of this combination therapy has also gained ground in BTC treatment. However, the effectiveness and safety profile remains largely elusive, especially in iCCA. As such, we evaluated the efficacy and safety of various anti-PD-1/L1 ICI, in combination with chemotherapy regimens compared to chemotherapy alone in iCCA patients with advanced, unresectable, and recurring tumors. Our findings showed that the combination of chemotherapy and anti-PD-1/L-1 has better outcomes with improved OS and PFS as well as ORR and DCR in patients with advanced iCCA compared to chemotherapy alone. This was also true in the various subgroups. Compared to the chemotherapy alone group, the chemotherapy plus anti-PD-1/L1 experienced more TRAEs. However, the TRAEs were manageable and tolerable.

Our findings demonstrated that the median OS in the combined therapy group is higher (11 months (95% CI 9.57–12.43)) versus (8 months (95% CI 6.34–9.64)) in the chemotherapy-alone group, with an HR of 0.53 (95% CI 0.30–0.94, *P = 0.008).* These findings are similar to other studies evaluating the efficacy of immunotherapy combined with chemotherapy in other types of advanced BTC. The phase 3 TOPAZ-1 trial, with a sample size of 198 patients in the combined therapy and 226 patients in the chemotherapy plus placebo, showed that the combination of GemCis plus durvalumab has better OS (12.8 months (95% CI, 11.1 to 14.0)) compared to chemotherapy plus placebo (11.5 months (95% CI, 10.1 to 12.5)) (16). Similarly, data from the KEYNOTE-966 trial which used pembrolizumab plus GemCis also showed higher OS (12.7 months (95% CI 11.5–13.6)) than GemCis plus placebo (10.9 months ((95% CI 9·9–11.6)) (15). The evaluation of PFS in chemotherapy alone and combined therapy shows that the combination of immunotherapy and chemotherapy increases the period of progression-free in patients with advanced iCCA. In our study, the PFS of combined therapy was 7 months while that of chemotherapy alone was 4 months. Comparatively, in another study, the combination of anti-PD-1 with chemotherapy also showed that combination therapy results in longer PFS (combined therapy 5.1 months; chemotherapy alone 2.4 months) (18). Together with our findings, these data suggest that combined therapy (anti-PD-1/L1 plus chemotherapy) produces positive prognosis outcomes for patients. Furthermore, our subgroup analysis reveals that various treatment regimens are likely to exhibit similar outcomes. This is independent of the use of either anti-PD-1 or anti-PD-L1. As such, chemotherapy can be combined with either anti-PD-1 or anti-PD-L1.

Our secondary outcomes, ORR (29.4%) and DCR (78.4%), in the combined group were significantly higher chemotherapy-alone group, 13.3%, and 73.3% respectively. In this study, 3 patients in the combined therapy achieved CR, which was similar to the findings by Danyang Sun et al. (18). Two of these patients were treated with chemotherapy plus durvalumab, this finding tallied with that of the TOPAZ-1 trial (16); thus, suggesting this combination regimen has a higher efficacy. The use of combination therapy is also likely to promote tumor disease downstaging, thereby increasing the likelihood of patients undergoing curative resection. In our study, 3 of the 12 patients who achieved PR underwent curative tumor resection following preoperative evaluation. Chemotherapy regimens have been previously demonstrated to promote tumor downsizing, including in iCCA (19, 20). We show that anti-PD-1 and/or anti-PD-L1 combined with chemotherapy still achieve similar outcomes, even in patients with inoperable iCCA. However, further prospective investigations are necessary to validate these observations.

In this study a total of 95.1% (77 of 81) patients experienced any grade TRAEs. The combination of chemotherapy with immunotherapy resulted in more but generally manageable TRAEs with the most severe TRAEs observed being grade 3–4, which accounted for 42%. Frequently observed TRAEs included leukopenia, thrombocytopenia, nausea, and anemia. In other studies, such as the TOPAZ-1 trial, similar adverse effects have been observed in combined therapy (16). Although our data shows comparable TRAEs to those in the TOPAZ-1 trail, their data showed 12 treatment-related deaths, while we observed none. The use of chemotherapy or combined therapy may result in immune-related adverse effects which are facilitated by myelosuppression and often cause discontinuation of treatment (12, 21, 22). Our observed immune-related TRAEs neither resulted in discontinuation of treatment nor required intervention, thus, suggesting the safety of combined therapy in iCCA treatment. However, close monitoring of patients is recommended during treatment.

### Limitations

Due to the lack of a prospective design, our study is subject to several limitations. The retrospective approach allows for the potential selection and recall bias. Furthermore, this study analyzed data from a single center and had a relatively limited sample size, as such, larger multicenter prospective investigations are necessary to consolidate our findings. This study did not assess a specific treatment regimen and a standardized dosage control, thus, potentially reducing the internal validity. Future investigations focusing on a single treatment regimen may be required. Since our study focused of advanced, unresectable and recurrent iCCA patients, our findings might not be generalized to other types of CCA. Finally, our study lacks pre-treatment testing for PD-L1 expression, tumor mutation burden (TMB), and microsatellite instability (MSI). These biomarkers may have a substantial impact on therapeutic outcomes. Although these limitations existed that questioned the validity of our findings, our study presents a real-world analysis that might be useful for future prospective studies.

## Conclusion

In conclusion, our findings suggest that combining a PD-1/L-1 inhibitor with chemotherapy in advanced iCCA treatment has higher anti-tumor efficacy compared to chemotherapy alone therapy. The enhanced survival outcomes together with tolerability to TRAEs in patients who received combined therapy underscores its potential as a therapeutic strategy for managing patients with advanced, unresectable, and recurrent iCCA in clinical settings. Combining anti-PD-1/L-1 agents with chemotherapy improves the survival of patients and can be used for tumor downsizing. The significance of our findings provides a basis for considering future large-scale and prospective research studies.

## Data availability statement

The raw data supporting the conclusions of this article will be made available by the authors, without undue reservation.

## Ethics statement

The studies involving humans were approved by The First Affiliated Hospital of Wenzhou Medical University ethics committee. The studies were conducted in accordance with the local legislation and institutional requirements. The ethics committee/institutional review board waived the requirement of written informed consent for participation from the participants or the participants’ legal guardians/next of kin because this study was conducted retrospectively.

## Author contributions

TM: Writing – review & editing, Writing – original draft, Visualization, Validation, Supervision, Software, Resources, Project administration, Methodology, Investigation, Formal Analysis, Data curation, Conceptualization. SH: Writing – review & editing, Writing – original draft, Supervision, Software, Project administration, Methodology, Investigation, Data curation, Conceptualization. HG: Writing – review & editing, Writing – original draft, Supervision, Project administration, Methodology, Investigation. YS: Writing – review & editing, Writing – original draft, Visualization, Validation, Supervision, Software, Resources, Project administration, Methodology, Investigation, Funding acquisition, Formal Analysis, Data curation, Conceptualization. EL: Writing – review & editing, Writing – original draft, Supervision, Project administration, Methodology, Investigation. JB: Writing – review & editing, Writing – original draft, Software, Project administration, Methodology, Investigation. AD: Writing – review & editing, Writing – original draft, Visualization, Investigation. BM: Writing – review & editing, Writing – original draft, Investigation. MB: Writing – review & editing, Writing – original draft, Investigation. MM: Writing – review & editing, Writing – original draft, Investigation. CB: Software, Writing – review & editing.

## Supplementary material

Supplementary Table 1 Treatment regimen for the Chemotherapy plus ant-PD-1/L1 group. *GP- Gemcitabine plus Cisplatin. *GEMOX- Gemcitabine and oxaliplatin

Supplementary Table 2 Treatment regimen for the chemotherapy group. *GS- Gemcitabine plus S-1. *FOLFIRINOX- oxaliplatin, irinotecan, and infusional fluorouracil.

## References

[1] Brindley PJ Bachini M Ilyas SI Khan SA Loukas A Sirica AE. Cholangiocarcinoma. Nat Rev Dis Primers. (2021) 7:65–. doi: 10.1038/s41572-021-00300-2 PMC9246479 34504109

[2] Banales JM Marin JJG Lamarca A Rodrigues PM Khan SA Roberts LR. Cholangiocarcinoma 2020: the next horizon in mechanisms and management. Nat Rev Gastroenterol Hepatology. (2020) 17:557–88. doi: 10.1038/s41575-020-0310-z PMC7447603 32606456

[3] Roskams TA Theise ND Balabaud C Bhagat G Bhathal PS Bioulac-Sage P. Nomenclature of the finer branches of the biliary tree: Canals, ductules, and ductular reactions in human livers. Hepatology. (2004) 39:1739–45. doi: 10.1002/hep.20130 15185318

[4] Kendall T Verheij J Gaudio E Evert M Guido M Goeppert B. Anatomical, histomorphological and molecular classification of cholangiocarcinoma. Liver Int. (2019) 39:7–18. doi: 10.1111/liv.14093 30882996

[5] Banales JM Cardinale V Carpino G Marzioni M Andersen JB Invernizzi P. Expert consensus document: Cholangiocarcinoma: current knowledge and future perspectives consensus statement from the European Network for the Study of Cholangiocarcinoma (ENS-CCA). Nat Rev Gastroenterol hepatology. (2016) 13:261–80. doi: 10.1038/nrgastro.2016.51 27095655

[6] Valle JW Kelley RK Nervi B Oh D-Y Zhu AX. Biliary tract cancer. Lancet. (2021) 397:428–44. doi: 10.1016/S0140-6736(21)00153-7 33516341

[7] Khan SA Tavolari S Brandi G. Cholangiocarcinoma: Epidemiology and risk factors. Liver Int. (2019) 39:19–31. doi: 10.1111/liv.14095 30851228

[8] Petrick JL Yang B Altekruse SF Van Dyke AL Koshiol J Graubard BI. Risk factors for intrahepatic and extrahepatic cholangiocarcinoma in the United States: A population-based study in SEER-Medicare. PloS One. (2017) 12:e0186643. doi: 10.1371/journal.pone.0186643 29049401 PMC5648218

[9] Florio AA Ferlay J Znaor A Ruggieri D Alvarez CS Laversanne M. Global trends in intrahepatic and extrahepatic cholangiocarcinoma incidence from 1993 to 2012. Cancer. (2020) 126:2666–78. doi: 10.1002/cncr.32803 PMC7323858 32129902

[10] Bertuccio P Malvezzi M Carioli G Hashim D Boffetta P El-Serag HB. Global trends in mortality from intrahepatic and extrahepatic cholangiocarcinoma. J Hepatology. (2019) 71:104–14. doi: 10.1016/j.jhep.2019.03.013 30910538

[11] Lamarca A Edeline J McNamara MG Hubner RA Nagino M Bridgewater J. Current standards and future perspectives in adjuvant treatment for biliary tract cancers. Cancer Treat Rev. (2020) 84:101936. doi: 10.1016/j.ctrv.2019.101936 31986437

[12] Valle J Wasan H Palmer DH Cunningham D Anthoney A Maraveyas A. Cisplatin plus gemcitabine versus gemcitabine for biliary tract cancer. New Engl J Med. (2010) 362:1273–81. doi: 10.1056/NEJMoa0908721 20375404

[13] Benson AB D’Angelica MI Abrams T Abbott DE Ahmed A Anaya DA. NCCN guidelines® Insights: biliary tract cancers, version 2.2023. J Natl Compr Cancer Network. (2023) 21:694–704. doi: 10.6004/jnccn.2023.0035 37433432

[14] Ueno M Ikeda M Morizane C Kobayashi S Ohno I Kondo S. Nivolumab alone or in combination with cisplatin plus gemcitabine in Japanese patients with unresectable or recurrent biliary tract cancer: a non-randomised, multicentre, open-label, phase 1 study. Lancet Gastroenterol hepatology. (2019) 4:611–21. doi: 10.1016/S2468-1253(19)30086-X 31109808

[15] Kelley RK Ueno M Yoo C Finn RS Furuse J Ren Z. Pembrolizumab in combination with gemcitabine and cisplatin compared with gemcitabine and cisplatin alone for patients with advanced biliary tract cancer (KEYNOTE-966): a randomised, double-blind, placebo-controlled, phase 3 trial. Lancet. (2023) 401:1853–65. doi: 10.1016/S0140-6736(23)00727-4 37075781

[16] Oh D-Y He AR Qin S Chen L-T Okusaka T Vogel A. Durvalumab plus gemcitabine and cisplatin in advanced biliary tract cancer. NEJM Evidence. (2022) 1. doi: 10.1056/EVIDoa2200015 38319896

[17] Rizvi S Khan SA Hallemeier CL Kelley RK Gores GJ. Cholangiocarcinoma - evolving concepts and therapeutic strategies. Nat Rev Clin Oncol. (2018) 15:95–111. doi: 10.1038/nrclinonc.2017.157 28994423 PMC5819599

[18] Sun D Ma J Wang J Han C Qian Y Chen G. Anti-PD-1 therapy combined with chemotherapy in patients with advanced biliary tract cancer. Cancer Immunology Immunother. (2019) 68:1527–35. doi: 10.1007/s00262-019-02386-w PMC6768892 31535160

[19] Kato A Shimizu H Ohtsuka M Yoshitomi H Furukawa K Takayashiki T. Downsizing chemotherapy for initially unresectable locally advanced biliary tract cancer patients treated with gemcitabine plus cisplatin combination therapy followed by radical surgery. Ann Surg Oncol. (2015) 22 Suppl 3:S1093–9. doi: 10.1245/s10434-015-4768-9 26240009

[20] Takayanagi R Takano S Sugiura K Yoshitomi H Furukawa K Takayashiki T. Successful radical surgical resection of initially unresectable intrahepatic cholangiocarcinoma by downsizing chemotherapy with gemcitabine plus cisplatin: a case report. Surg Case Rep. (2017) 3:116. doi: 10.1186/s40792-017-0395-y 29164423 PMC5698235

[21] Mitsudomi T Morita S Yatabe Y Negoro S Okamoto I Tsurutani J. Gefitinib versus cisplatin plus docetaxel in patients with non-small-cell lung cancer harbouring mutations of the epidermal growth factor receptor (WJTOG3405): an open label, randomised phase 3 trial. Lancet Oncol. (2010) 11:121–8. doi: 10.1016/S1470-2045(09)70364-X 20022809

[22] Lin J Yang X Long J Zhao S Mao J Wang D. Pembrolizumab combined with lenvatinib as non-first-line therapy in patients with refractory biliary tract carcinoma. Hepatobiliary Surg Nutr. (2020) 9:414–24. doi: 10.21037/hbsn-20-338 PMC7423565 32832493

