# Supplementary material for: Real-world outcomes of chemotherapy plus immune checkpoint inhibitors versus chemotherapy alone in advanced, unresectable, and recurrent intrahepatic cholangiocarcinoma
Source: Front Immunol. 2024 May 23;15:1390887. doi: 10.3389/fimmu.2024.1390887 (PMC11153733; doi:10.3389/fimmu.2024.1390887)
Supplement: Supplementary Table 1 — Treatment regimen for the Chemotherapy plus ant-PD-1/L1 group. *GP- Gemcitabine plus Cisplatin. *GEMOX- Gemcitabine and oxaliplatin [file Table_1.docx]

Supplementary data

**Table S1.**

| **Patient** | **Regimen** | **Number of cycles** | **Patient** | **Regimen** | **Number**  **of cycles** |
| --- | --- | --- | --- | --- | --- |
| 1 | GP + Durvalumab | 8 | 27 | GP + Pembrolizumab | 8 |
| 2 | GP + Camrelizumab | 8 | 28 | GP +Durvalumab | 5 |
| 3 | GP + Camrelizumab | 12 | 29 | GP + Pembrolizumab | 7 |
| 4 | GEMOX + Pembrolizumab | 3 | 30 | GEMOX + Durvalumab | 5 |
| 5 | GEMOX + Camrelizumab | 2 | 31 | GEMOX + Durvalumab | 7 |
| 6 | GP + Pembrolizumab | 5 | 32 | GP + Durvalumab | 7 |
| 7 | GEMOX + Camrelizumab | 5 | 33 | GEMOX + Durvalumab | 10 |
| 8 | GP + Pembrolizumab | 6 | 34 | GP + Durvalumab | 5 |
| 9 | GP +Durvalumab | 8 | 35 | GP + Durvalumab | 4 |
| 10 | GEMOX + Durvalumab | 7 | 36 | GP + Pembrolizumab | 3 |
| 11 | GP + Pembrolizumab | 6 | 37 | GP +Durvalumab | 4 |
| 12 | GEMOX + Durvalumab + tegafur | 4 | 38 | GEMOX + Durvalumab | 13 |
| 13 | GP + Pembrolizumab | 8 | 39 | GP + Pembrolizumab | 3 |
| 14 | GP + Camrelizumab | 7 | 40 | GP + Pembrolizumab | 7 |
| 15 | GP + Pembrolizumab | 9 | 41 | GEMOX + Durvalumab | 8 |
| 16 | GP +Durvalumab | 4 | 42 | GP + Pembrolizumab | 3 |
| 17 | GEMOX + Durvalumab | 3 | 43 | GP + Durvalumab+ tegafur | 7 |
| 18 | GEMOX + Durvalumab | 6 | 44 | GP + Durvalumab | 4 |
| 19 | GP + Pembrolizumab | 3 | 45 | GP + Durvalumab | 5 |
| 20 | GP +Durvalumab | 14 | 46 | GP + Durvalumab | 3 |
| 21 | GP + Camrelizumab | 3 | 47 | GP +Durvalumab | 8 |
| 22 | GP + Pembrolizumab | 2 | 48 | GP + Pembrolizumab | 5 |
| 23 | GP +Durvalumab | 5 | 49 | GP + Pembrolizumab | 2 |
| 24 | GEMOX + Durvalumab | 5 | 50 | GP + Camrelizumab | 3 |
| 25 | GP + Camrelizumab | 6 | 51 | GP + Pembrolizumab | 4 |
| 26 | GP +Durvalumab | 5 |  |  |  |

**Table S2.**

| **Patient** | **Regimen** | **Number of cycles** | **Patient** | **Regimen** | **Number of cycles** |
| --- | --- | --- | --- | --- | --- |
| 1 | Gemcitabine + Oxaliplatin | 6 | 16 | Gemcitabine + Cisplatin | 5 |
| 2 | Gemcitabine + Cisplatin | 4 | 17 | FOLFIRINOX | 6 |
| 3 | GS | 8 | 18 | Gemcitabine + Oxaliplatin | 3 |
| 4 | GS | 3 | 19 | GS | 7 |
| 5 | Gemcitabine + Oxaliplatin | 2 | 20 | Gemcitabine + Oxaliplatin | 2 |
| 6 | Gemcitabine + Cisplatin | 12 | 21 | Gemcitabine + Cisplatin | 5 |
| 7 | FOLFIRINOX | 6 | 22 | Gemcitabine + Cisplatin | 6 |
| 8 | Gemcitabine + Cisplatin | 3 | 23 | Gemcitabine + Cisplatin | 6 |
| 9 | Gemcitabine + Cisplatin | 2 | 24 | Gemcitabine + Oxaliplatin | 12 |
| 10 | Gemcitabine + Cisplatin | 5 | 25 | Gemcitabine + Cisplatin | 4 |
| 11 | FOLFIRINOX | 8 | 26 | Gemcitabine + Cisplatin | 9 |
| 12 | GS | 6 | 27 | FOLFIRINOX | 7 |
| 13 | Gemcitabine + Oxaliplatin | 8 | 28 | GS | 7 |
| 14 | Gemcitabine + Cisplatin | 3 | 29 | Gemcitabine + Oxaliplatin | 5 |
| 15 | Gemcitabine + Oxaliplatin | 5 | 30 | Gemcitabine + Cisplatin | 3 |

Legends.

***Table S1.*** *Treatment regimen for the Chemotherapy plus ant-PD-1/L1 group*

**GP- Gemcitabine plus Cisplatin*

**GEMOX-* *Gemcitabine and oxaliplatin*

***Table S2.*** *Treatment regimen for the chemotherapy group*

**GS-* *Gemcitabine plus S-1*

**FOLFIRINOX-* *oxaliplatin, irinotecan, and infusional fluorouracil*
